# The microbiome of kidney stones and urine of patients with nephrolithiasis

**DOI:** 10.1007/s00240-022-01403-5

**Published:** 2023-01-04

**Authors:** Ursula Lemberger, Petra Pjevac, Bela Hausmann, David Berry, Daniel Moser, Victoria Jahrreis, Mehmet Özsoy, Shahrokh F. Shariat, Julian Veser

**Affiliations:** 1 Research Laboratory, Department of Urology, Medical University of Vienna, Waeringerguertel 18-20, 1090 Vienna, Austria; 2 Joint Microbiome Facility of the Medical University of Vienna and the University of Vienna, Vienna, Austria; 3 Division of Microbial Ecology, Department of Microbiology and Ecosystem Science, Centre for Microbiology and Environmental Systems Science, University of Vienna, Vienna, Austria; 4 Department of Laboratory Medicine, Medical University of Vienna, Vienna, Austria; 5 Departments of Urology, Weill Cornell Medical College, New York, USA; 6 Department of Urology, University of Texas Southwestern, Dallas, TX USA; 7 Department of Urology, Second Faculty of Medicine, Charles University, Prague, Czech Republic; 8 Institute for Urology, University of Jordan, Amman, Jordan

**Keywords:** Nephrolithiasis, Microbiome, Kidney stones, Metabolic syndrome, 16S rRNA gene amplicon sequencing

## Abstract

The incidence of nephrolithiasis is rising worldwide. Although it is a multifactorial disease, lifestyle plays a major role in its etiology. Another considerable factor could be an aberrant microbiome. In our observational single-center study, we aimed to investigate the composition of bacteria in kidney stones and urine focusing on patients with features of metabolic syndrome. Catheterized urine and kidney stones were collected prospectively from 100 consecutive patients undergoing endoscopic nephrolithotomy between 2020 and 2021 at our clinic. Microbiome composition was analyzed via 16S rRNA gene amplicon sequencing. Detection of bacteria was successful in 24% of the analyzed kidney stones. These patients had a prolonged length of stay compared to patients without verifiable bacteria in their stones (2.9 vs 1.5 days). Patients with features of metabolic syndrome were characterized by kidney stones colonized with classical gastrointestinal bacteria and displayed a significant enrichment of *Enterococcaceae* and *Enterobacteriaceae.* Stones of patients without features of metabolic syndrome characterized by *Ureaplasma* and *Staphylococcaceae*. Patients with bacteria in their kidney stones exhibit a longer length of stay, possibly due to more complex care. Patients presenting with features of metabolic syndrome displayed a distinct stone microbiome compared to metabolically fit patients. Understanding the role of bacteria in stone formation could enable targeted therapy, prevention of post-operative complications and new therapeutic strategies.

**Supplementary Information:**

The online version contains supplementary material available at 10.1007/s00240-022-01403-5.

## Introduction

Nephrolithiasis is a rising problem globally, with the lifetime incidence having increased from 3% in the 1970s to 10% in Europe, 14% in North America and 20% in Saudi Arabia [1]. In parallel, the rate of stone recurrence has increased with almost 50% of stone patients experiencing an additional stone event within 5 years [2]. In the western world, both sexes are now almost equally affected. However, the prevalence for stone disease (SD) increased with 22.0% in females, while it stayed almost constant in men [3, 4].

For a long time, urinary supersaturation with calcium and oxalate beyond their solubility was considered the main cause of stone formation [5]. Recent studies showed, that recurrent stone formers do not necessarily present with urinary accumulation of calcium oxalate (CaOx) or calcium phosphate (CaPhos). In fact, there is no significant difference in urine chemistry of recurrent stone builders and healthy controls, suggesting that chemical supersaturation alone is not sufficient to explain stone formation [6].

Dysbiosis of intestinal, skin and mucosal microbiomes has been associated with different maladies. Whether urinary dysbiosis is a driver of stone disease, or stone formation rather promotes bacterial imbalance of the urological tract remains to be clarified.

### The urogenital microbiome

Bacteria are essential for urogenital homeostasis, and imbalances in the microbiome can contribute to various urological diseases, such as urinary tract infection, voiding disorders, but also tumorigenesis.

Multiple bacterial species have been shown to be involved in stone formation, or might contribute to the prevention of nephrolithiasis [7, 8]. However, no specific bacterial species has been identified to be singularly responsible for stone formation so far. Therefore, we hypothesized that an imbalance in the urinary microbiome is the main driver for nephrolithiasis and recurrent stone disease. Besides long-time antibiotic therapy, body weight and imbalances in metabolism are associated with microbial dysbiosis [9]. Hence, we aimed to analyze the microbiome in stones and urine of patients with and without features of metabolic syndrome, to further investigate connections between the microbiome and the most common co-morbidity of nephrolithiasis.

## Materials and methods

### Patient population

Catheterized urine and kidney stones were prospectively collected intraoperatively from 100 consecutive patients undergoing endoscopic nephrolithotomy between January 2020 and February 2021 at our clinic. The study was approved by the Ethics committee of the medical University of Vienna (2093/2019), and all patients signed an informed consent. Patients receiving antibiotic therapy within six months prior to surgery, presenting with an acute urinary tract infection, anatomic anomalies of the kidney and urinary tract, autoimmune and gastrointestinal disease, genetic diseases associated with SD and an age under 18 years were not sampled.

### Sample collection

Urine (15–50 ml) and kidney stones were collected and processed according the standardized consensus agreement for microbiome studies for nephrolithiasis [10]. A fragment of all extracted stones was sent for lithography via dust X-ray diffractometry (XRPD) to the Institute for Mineralogy and Petrography, University of Innsbruck, Austria. Baseline clinical data, comorbidities, stone composition and results from pre-surgical urine cultures were documented.

Mid-stream voided urine of volunteers without a history of stone disease served as reference and benchmark of a sex, age and BMI-adjusted group. Exclusion criteria and processing of urine were the same as for the study group and according the guidelines [10].

### Sample processing

Stones were pulverized in a blender. DNA was extracted using the #47,016 DNA-EASY PowerSoil Kit (Qiagen). For DNA extraction from pelleted urine samples, the #51,704 QIAAMP DNA Microbiome Kit (Qiagen) was used. Both were handled according to the manufacturer’s instructions.

### 16S rRNA gene amplification and sequencing

16S rRNA gene amplification, sequencing as well as raw data processing, was performed by the Joint Microbiome Facility (JMF) (project ID JMF-2102-05) via an Illumina MiSeq-based highly multiplexed gene amplicon sequencing workflow. Amplification and sequencing of the V3–V4 hypervariable region of the bacterial 16S rRNA gene were performed with a two-step PCR approach, as described previously [11].

### Statistical analysis

Amplicon sequence variants (ASVs) were inferred using the DADA2 R package v1.20 applying the recommended workflow [12, 13]. FASTQ reads 1 and 2 were trimmed at 230 nucleotides with allowed expected errors of 4 and 6, respectively. ASV sequences were subsequently classified using DADA2 and the SILVA database SSU Ref NR 99 release 138.1 using a confidence threshold of 0.5. ASVs classified as eukaryotes, mitochondria, or chloroplast were removed [14]. Downstream analyses were performed using R v4.2 and Bioconductor v3.15 packages TreeSummarizedExperiment v2.4[15], mia v1.4 (https://github.com/microbiome/mia), vegan v2.6.2 (https://CRAN.R-project.org/package=vegan), phyloseq v1.40[16], microbiome v1.18[15], microViz v0.9.2[17], DESeq2 v1.34 [18]. Alpha diversity was calculated on verified data (500 reads per sample) using vegan and mia. Beta diversity was calculated by performing a PCoA with an Aitchison distance matrix using microViz. The difference in per-group centroids was tested with a PERMANOVA on an Aitchison distance matrix using vegan and microViz. Pairwise differential abundance testing was performed using DESeq2 with alpha = 0.05 and otherwise default parameters after adding a pseudo-count of 1 to the data.

LOS and data derived from urine cultures were analyzed via parametric *t*-test using GraphPad Prism8. Report of categorical variables included frequencies and proportions. Continuous variables were reported as means and standard deviation.

### Data availability

16S rRNA gene amplicon sequencing data were deposited in the Sequence Read Archive under the BioProject accession number PRJNA884967. https://www.ncbi.nlm.nih.gov/sra/?term=PRJNA884967.

## Results

Catheterized urine and kidney stones from 72 male (72%), and 28 female (28%) patients were collected intraoperatively. Voided urine of 20 volunteers (10 males and 10 females) served as healthy reference group. All patient characteristics are shown in Supplementary Fig. 1A. In the study cohort, 18 different combinations of stone types were identified via XRPD. The most prevalent stone type was CaOx monohydrate (COM, Whewellite) present in 71.4% of female and 33.3% of male patients, followed by mixed stones of Whewellite/Apatite (10.7%) in females and Whewellite/CaOx dihydrate and monohydrate (COD, Weddellite) in male patients (18.1%) (Supplementary Fig. 1B).

**Fig. 1:**
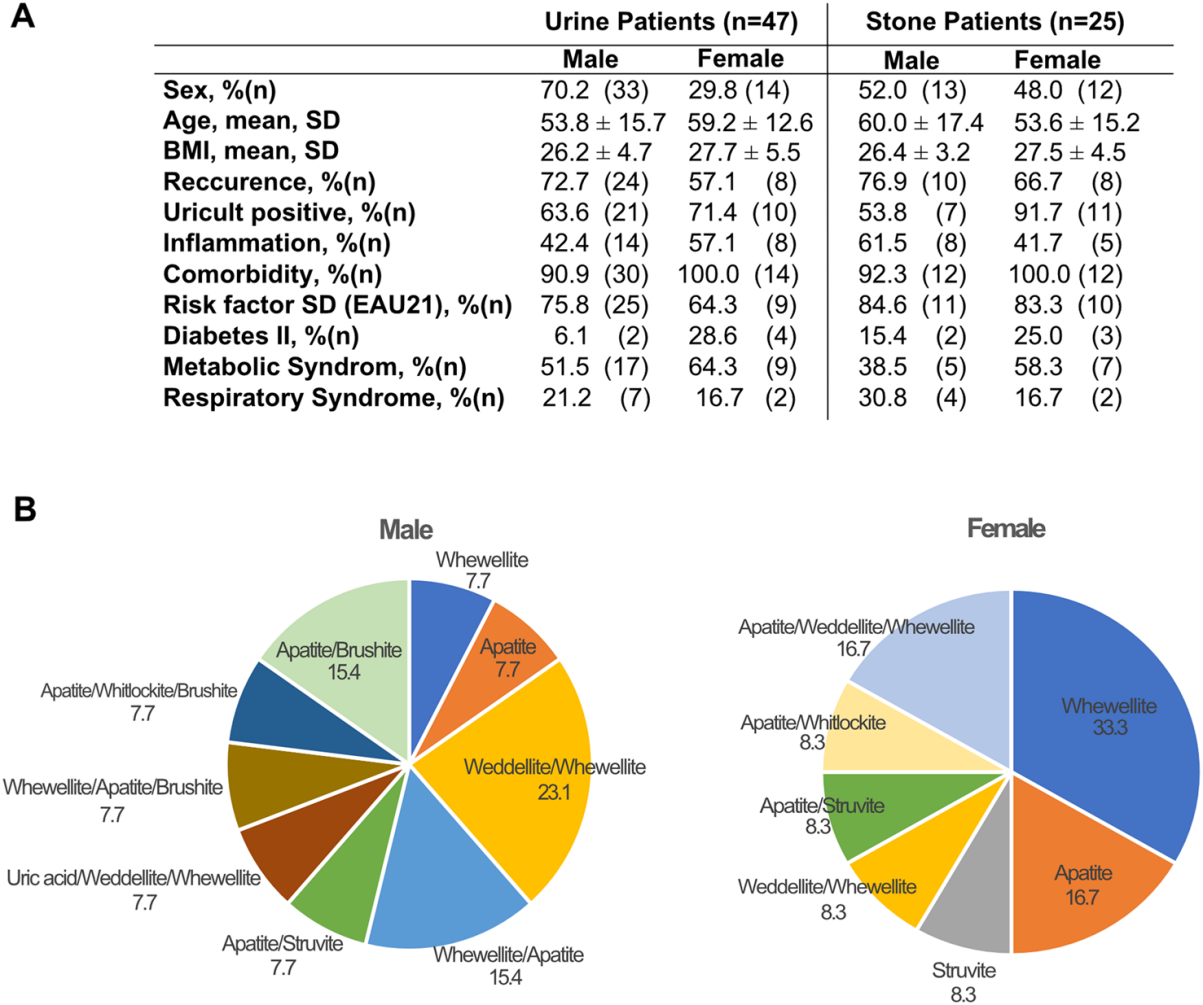
Clinical Data Patient data of urine samples and stone samples (**A**) with > 500 reads. Distribution of stone types per sex in % (**B**)

Due to the low microbial biomass in some samples, 16S rRNA gene amplification was not successful in all collected samples. After quality control and contamination filtering, we only used a sub-cohort of samples for further analysis, where the minimal read number surpassed 500 reads. Urine samples from 33 male (45.8%) and 14 female (50%) patients met the filter criteria, while only 13 stones from male and 12 stones from female patients could be included (18.1 and 42.9%, respectively). The voided urine of all volunteers passed the quality control (Supplementary Fig. 1A). Notably, clinical data in the successfully sequenced samples are similar to the initial cohort (Fig. 1A, B). Finally, for 5 male (6.9%) and 7 female (25%) patients, microbiome data could be retrieved from a full sample set (Supplementary Fig. 1A).

Almost all stone types had a verifiable microbiome. However, all pure uric acid stones (12.7% of all stones) had to be excluded, since none of them displayed > 500 reads (mean = 37.5 reads). The highest read numbers were observed in Apatite and Apatite/Weddellite/Whewellite stones.

### Bacterial community composition and its variation between controls and cases

In total, 828 different taxa, 728 different genera and 240 species were identified.

**Fig. 2:**
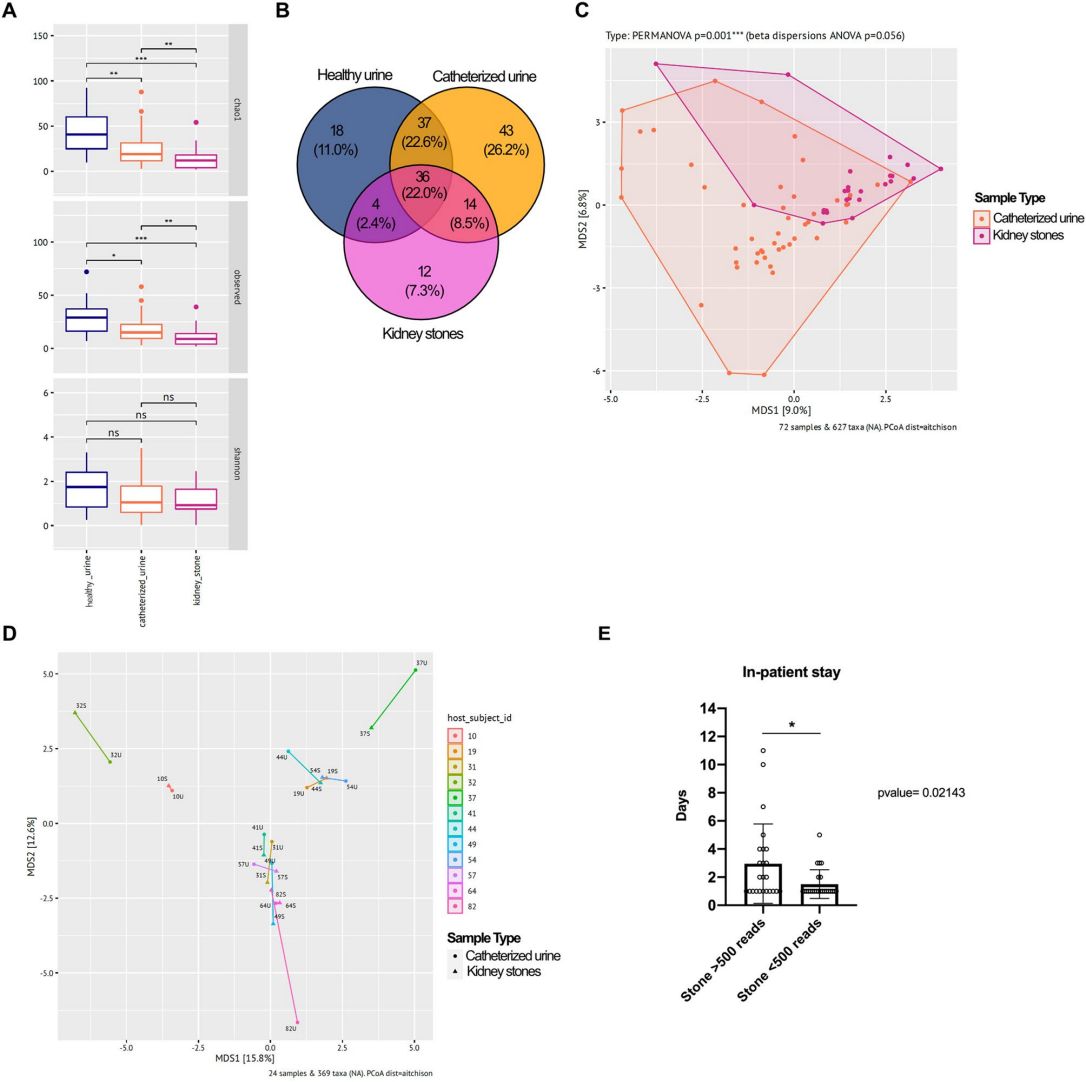
Microbiome analysis by sample type Alpha diversity calculated by Chao, observed and Shannon Index (**A**), Venn diagram of mutual and distinct genera per sample type (**B**). PCoA revealed significant difference between kidney stones and urine (*p*-value = 0.001***; **C**) but a close relation between individual pairs (**D**), highlighting the similarities of bacterial populations in stones (S) and respective urine (U). Patients with > 500 reads in stone material displayed a prolonged post-operative stay compared to patients with < 500 reads per stone (2.9 vs 1.5 days, *p* = 0.021*, E)

The microbiome of voided urine samples in the reference cohort without kidney stones exhibited a higher alpha diversity (Chao1 and observed ASVs) than catheterized patient urine samples, while kidney stone samples were the least diverse. The species richness was significantly higher in voided urine samples from healthy volunteers compared to catheterized urine of SD patients (Chao1: *p*-value 0.0072) and kidney stones (Chao1: *p*-value 0.00054). However, no significant difference in diversity based on the Shannon Index was determined between the three groups (Fig. 2A). The higher diversity and bacterial load in voided healthy urine is likely related to the sampling approach, since voided mid-stream urine can also include vaginal and skin microbiome. Bacterial 16S rRNA gene sequences affiliated to 18 genera were solely found in voided urine samples, while 43 genera were only detected in catheterized urine and 12 only in kidney stone samples (Fig. 2B). Thirty-six genera were found in all three sample groups, while urine derived from SD patients and volunteer urine harbored representatives of 37 mutual genera. The lowest alignment was between stones and control urine, where representatives of only four common genera were identified.

Beta diversity of stone microbiomes and respective catheterized patient urine (PERMANOVA *p* = 0.001) displayed a significant heterogeneity in variance indicated by beta dispersion (ANOVA *p* = 0.00056), Fig. 2C). However, we detected no significant difference in bacterial diversity between stone and urine samples in the sub-cohort (Supplementary Fig. 1A), where matching samples of kidney stone and respective urine were available. In fact, in the majority of cases, matching stone and urine samples displayed the lowest degree of dissimilarity (Fig. 2D).

Interestingly, patients with verifiable stone microbiome displayed a prolonged post-operative stay compared to patients where the stone microbiome was below 500 reads (2.9 vs 1.5 days, *p* = 0.021) (Fig. 2E). Reasons for the extended in-patient stay were bleeding (16.6%), fever (16.6%) and/or pain (12.5%).

## Significant differences in relative abundance of bacterial general between sample types

**Fig. 3:**
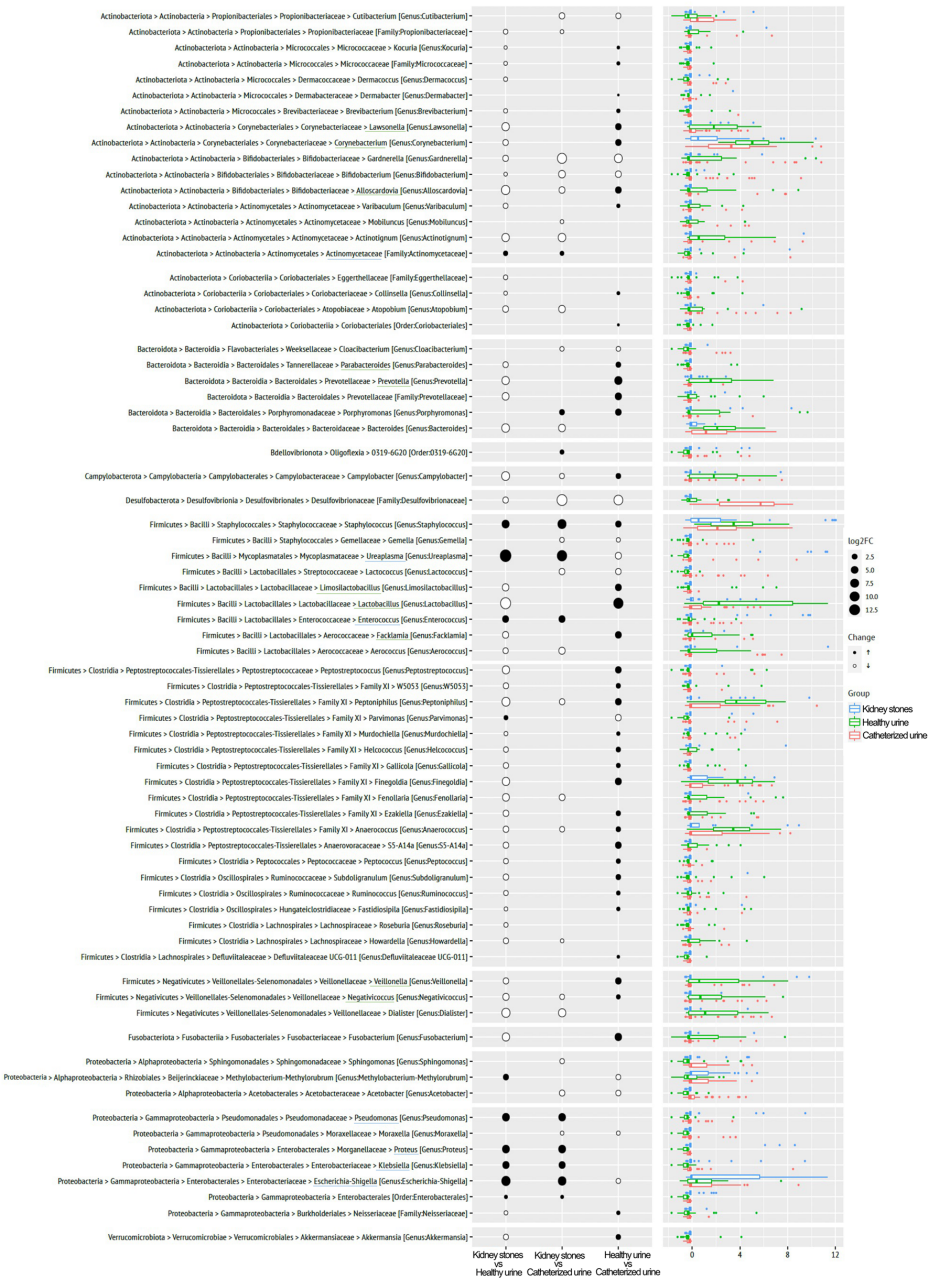
Differential relative abundance analysis by sample types Comparison of significantly deregulated genera per sample type. Black dots indicate increase, white a decrease of genus of the top compared to the bottom cohort, dot size illustrates the taxon abundance. Green underlined genera were predominantly found in healthy urine, blue underlined were significantly upregulated in kidney stones compared to catheterized or voided urine

We further analyzed the differential relative abundance of microbial taxa, grouped on genus level, between stones, patient urine and urine from healthy volunteers. DeSeq2 analysis revealed 72 significantly differentially abundant genera among the three sample groups (Fig. 3). Comparing the microbiome of stones to corresponding patient urine or healthy urine respectively, we determined that *Ureaplasma*, *Escherichia–Shigella* (log2FC: 12.5), *Pseudomonas*, *Proteus*, *Klebsiella*, *Enterococcus* (log2FC: 10.0), and other *Enterobacterales* and *Enterococcaceae* (highlighted in blue) displayed a significantly increased relative abundances in stone samples when compared to both types of urine samples.

In voided urine from volunteers without nephrolithiasis, several genera belonging to *Firmicutes*, such as *Finegoldia*, *Anaerococcus*, *Peptoniphilus* and *Veillonella*, were more abundant. *Corynebacteriaceae* (*Lawsonella*, *Corynebacterium*), B*ifidobacteriaceae* (*Alloscardovia*, *Coriobacteriaceae*) and *Lactobacillaceae* (*Lactobacillus*, *Limosilactobacillus*, *Facklamia*) were also more abundant in healthy mid-stream urine (highlighted in green), compared to patient urine or stone samples.

## Patients with features of metabolic syndrome display a distinct microbiome

We further investigated the correlation of dysbiosis in SD patients with clinical data.

**Fig. 4:**
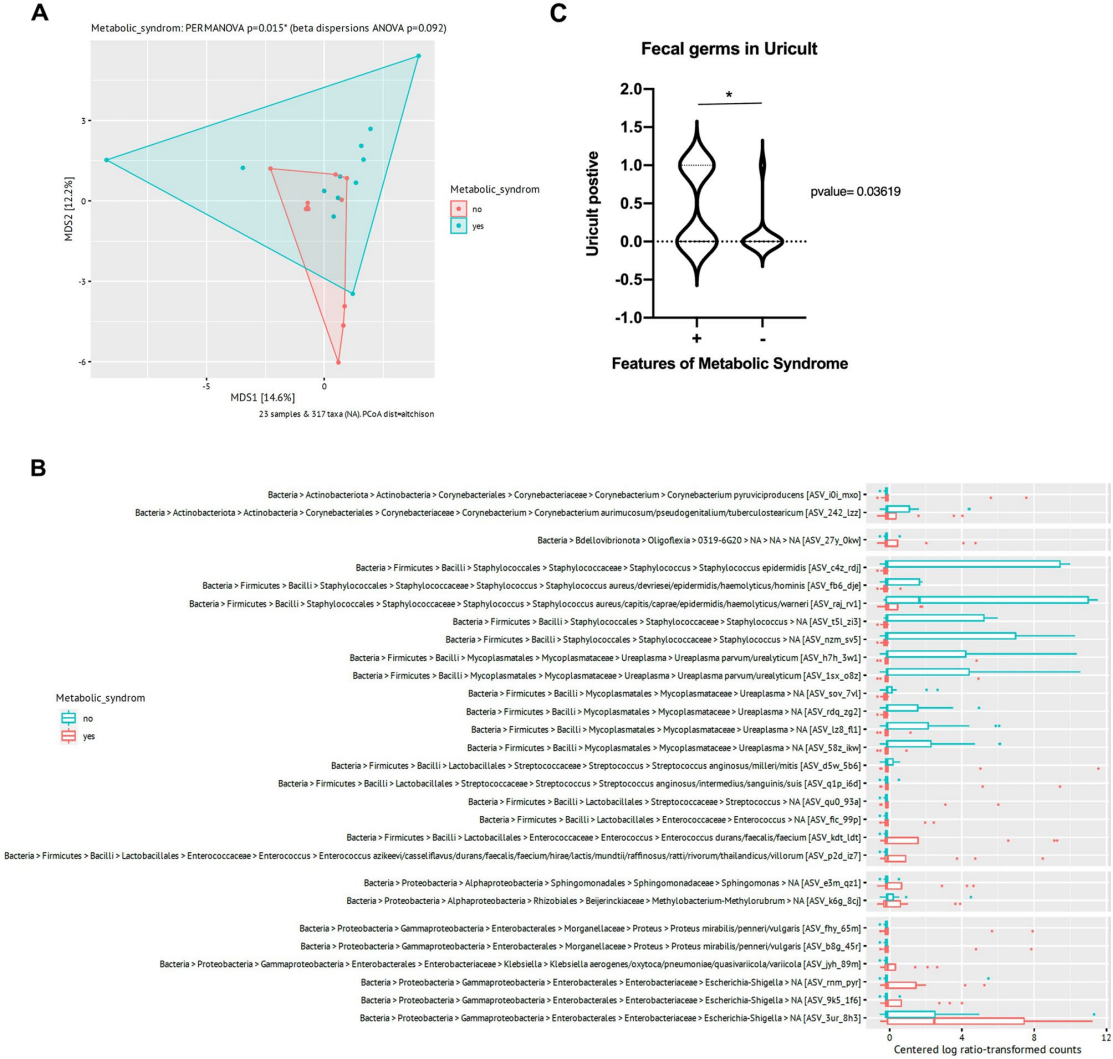
Patients with features of metabolic syndrome display a distinct microbiome (PERMANOVA *p*-value = 0.015*, **A**) and an enrichment of classical gastrointestinal tract-associated microorganisms in stones, but lack *Ureaplasma* and *Staphylococcaceae* (**B**), which are highly abundant in stones of patients with no FMS. Also, urine cultures of patients with features of metabolic syndrome were significantly more often positive for fecal bacteria (*E. faecalis* and *E. coli*), (*p*-value = 0.0361*, **C**)

Patients were clustered to the cohort “features of metabolic syndrome” (FMS), when at least two of the following morbidities were exhibited: BMI > 30, Type 2 diabetes, dyslipidemia and arterial hypertension. Interestingly, PCoA analysis revealed a significant difference in the stone microbiome in patients with FSM compared to those without FMS (*p* = 0.015) (Fig. 4A). In kidney stones derived from patients with FSM, the genera *Escherichia–Shigella*, *Enterococcus*, *Sphingomonas* and *Klebsiella* were significantly more relatively abundant. Stones retrieved from patients without FMS were characterized by increased relative abundance of different *Staphylococcus* species and *Ureaplasma* instead, which were almost absent in stones from patients with FMS (Fig. 4B).

Additionally, patients with FMS displayed a significantly increased relative abundance of classical gastrointestinal tract-associated microorganisms, such as *E. coli*, and *Enterococcus faecalis* in pre-surgical urine cultures (> 10^3^ colony-forming units/ml) compared to SD patients without FMS (*p* = 0.0362) (Fig. 4C).

No significant difference was found regarding recurrence of SD, and other clinical parameters, when compared to the cohort without the respective condition (data not shown). Given the small number of patients with the same stone type fulfilling the quality criteria, we also found no significant similarities in the microbiome within a certain stone type (Supplementary Fig. 2).

## Discussion

We performed the so far largest prospective study investigating the composition of kidney stones and urine microbiome in patients with nephrolithiasis. Our analyses lead to several important findings. First, the highest amounts of confidently detectable bacterial colonization were found in Apatite and Apatite/CaOx/CaPhos stones, although these stones are classified as “non-infection stones” in the EAU guidelines [19]. Second, we observed that the microbiome of stones is well reflected in the corresponding patients’ urine sample. Thus, the analysis of the microbial community composition of catheterized urine samples may help to guide therapy decisions for tailored antibiosis, to avoid complications after stone removal but also antimicrobial overtreatment. Moreover, our analysis revealed, that stones display an increased relative abundance of distinct microorganisms, classically associated with the gastrointestinal microbiome, but also known to be pathogenic [20].

The systemic change of public health, with increasing cases of severe obesity, raises enormous medical expenditure and imposes new challenges in patient care. Nephrolithiasis is a well-known co-morbidity of obesity, and metabolic syndrome and diabetes have been considered as risk factors for kidney stone formation for more than 20 years [21]. For a long time, hyper-nutrition and unilateral diet, resulting in a supersaturation of carbonates, considered mainly as the underlying reasons. Moreover, obese persons are characterized by hyperinsulinemia, which is associated with increased intestinal absorption and renal excretion of calcium [22]. However, besides resulting in chemical supersaturation, nutrition has a strong effect on the gut microbiome itself, and consequently also on urogenital microbial populations [23]. Consistent with previous reports, we found significant dysbiosis in patients with SD, characterized by an enrichment of classical gastrointestinal microorganisms in urine and kidney stones [23]. While others focused on dysbiosis of the gut microbiome in SD, we were able to confirm this observation also in stone and urine of patients with nephrolithiasis [24]. We showed that the bacterial composition in stones of patients with features of metabolic syndrome is characterized by increased abundance of gastrointestinal microorganisms, such as *E. coli*,* Shigella*,* Klebsiella*,* Enterococcaceae*,* Proteus* and *Sphingomonas*, while SD patients without FMS display a higher relative abundance of *Staphylococcaceae* and *Ureaplasma.* This supports the study from Chen et al. where an increased abundance of *Escherichia–Shigella*, *Klebsiella*, *Enterococcus* and *Aerococcus*, but a reduction *of Prevotella* and *Lactobacillus* in patients with type II diabetes and lower urinary tract symptoms (LUTS) was described [25]. The origin of these classical fecal opportunistic pathogens and commensals is not easily explained. It is possible that poor diet and the enrichment of carbohydrates promote the colonization of certain bacteria, while overgrowing others.

We were not able to demonstrate that the accumulation of a specific bacterial genus or combination of bacteria promotes the formation of a particular type of kidney stone. However, we showed that the incidence of pathogenic *Enterobacteriaceae* was high in all stone types, proving that besides struvite, CaOx and CaPhos stones are also associated with these bacteria. Barr-Baere et al. also identified *E. coli* and other bacteria of the family *Enterobacteriaceae* as the most prevalent uro-pathogen in pediatric urinary tract infections accompanied by CaOx stones [26]. Taking advantage of a murine model for CaOx nephropathy, they showed increased CaOx deposition in mice after transurethral inoculation with *E. coli* compared to CaOx nephropathy alone. The underlying mechanism for enhanced stone formation was both the adhesion of bacterial metabolites to crystals and increased CaOx crystal aggregation [26].

Furthermore, we hypnotized that patients with recurrent SD display an aberrant microbiome compared to patients with primary SD. Reasons therefore might be previous interventions, transporting bacteria from skin, genitals, urethra and bladder up to the renal pelvis. Moreover, antibiosis and other therapeutics can also sustainably change the bacterial composition of the urogenital tract, resulting in dysbiosis and therefore recurrent stone formation. Surprisingly, we were not able to detect significant divergence in stone and urine microbiomes of recurrent SD patients compared to primary SD patients. However, the five patients with the highest read sequence yield per stone (> 100.000 reads/stone) had multiple stone removals within the study period, with increasing read numbers in stones but not in urine. Notably, all of them had Apatite or Apatite mix stones.

Interestingly, patients with verifiable bacteria in kidney stones displayed a prolonged LOS due to different post-operative complications. Similarly, Wagenius et al. demonstrated, that patients with positive stone cultures harbor a greater risk for post-operative urosepsis, than those with just positive urine culture [27]. So the evidence of pathogens in the kidney is a tremendous risk for infectious complications.

The major limitation of our study was the limited number of patients for which sufficient microbial biomass was retrievable, impairing statistic correlations.

 We also want to address the problem of a suitable control group in urological microbiome research. Catheterized urine from healthy individuals would be the most suitable control to reveal disease-related dysbiosis. Since this cohort was not available due to ethical reasons, we investigated the voided mid-stream urine of volunteers without prior stone disease. Hence, we do not consider the data obtained from voided urine as a control, but rather as a benchmark and reference, to detect bacteria unique to patients with stone disease.

As previously described, we observed a sex-specific voided urine microbiome in healthy men and women (*p*-value 0.002, Supplementary Fig. 3A), but not in catheterized urine of patients with SD (Supplementary Fig. 3B) [28, 29]. Moreover, we observed no difference in stone microbiome between man and women. This result is certainly influenced by the approach of urine collection, since voided urine represents a combination of bladder, urethral, and perimeatal microbiota. Therefore, with the available data, we are not able to conclude if the difference of sexes between healthy urine and urine form patients with stone disease is due to dysbiosis.

Additional studies including whole metagenome sequencing are required to improve our understanding of the involvement and role of bacteria in kidney stone disease. Moreover, concordant analyses of patients` voided and catheterized urine, stones and gut microbiome are necessary to illuminate the extent of dysbiosis and interactions of the microbes to prevent stone formation, tailor antibiotic therapy and circumvent postsurgical complications.

## Conclusion

The current study showed that patients presenting with features of metabolic syndrome displayed a distinct stone microbiome compared to metabolically fit patients. They displayed a significant enrichment of classical gastrointestinal bacteria, such as *E. coli*,* Shigella*,* Klebsiella*,* Enterococcaceae*,* Proteus* and *Sphingomonas.* Stones of patients without features of metabolic syndrome were characterized by *Ureaplasma* and *Staphylococcaceae*.

Generally, patients with bacteria in their kidney stones exhibit a longer length of stay, presumably due to more complex care.

### Supplementary Information

Below is the link to the electronic supplementary material.

Supplementary file1 Supplementary Fig. 1 Table with clinical data of all collected patients with nephrolithiasis and all patients with stone and urine sample >500 reads (**A**). Distribution of all collected stone types per sex in % (**B**) (TIFF 1100 KB)

Supplementary file2 Supplementary Fig. 2 PCoA of Stone type and associated microbiome. Kidney stones with the same chemical composition display no significant similarities in their microbiome (TIFF 688 KB)

Supplementary file3 Supplementary Fig. 3 Correlation of urine microbiome and sex. In men and women without stone disease urine samples displayed a significant difference in microbiome composition (**A**), while there is no difference between sexes in patients with nephrolithiasis (**B**) (TIFF 782 KB)
