# Supplementary figures and images for: The microbiome of kidney stones and urine of patients with nephrolithiasis
Source: Urolithiasis. 2023 Jan 4;51(1):27. doi: 10.1007/s00240-022-01403-5 (PMC9810570; doi:10.1007/s00240-022-01403-5)

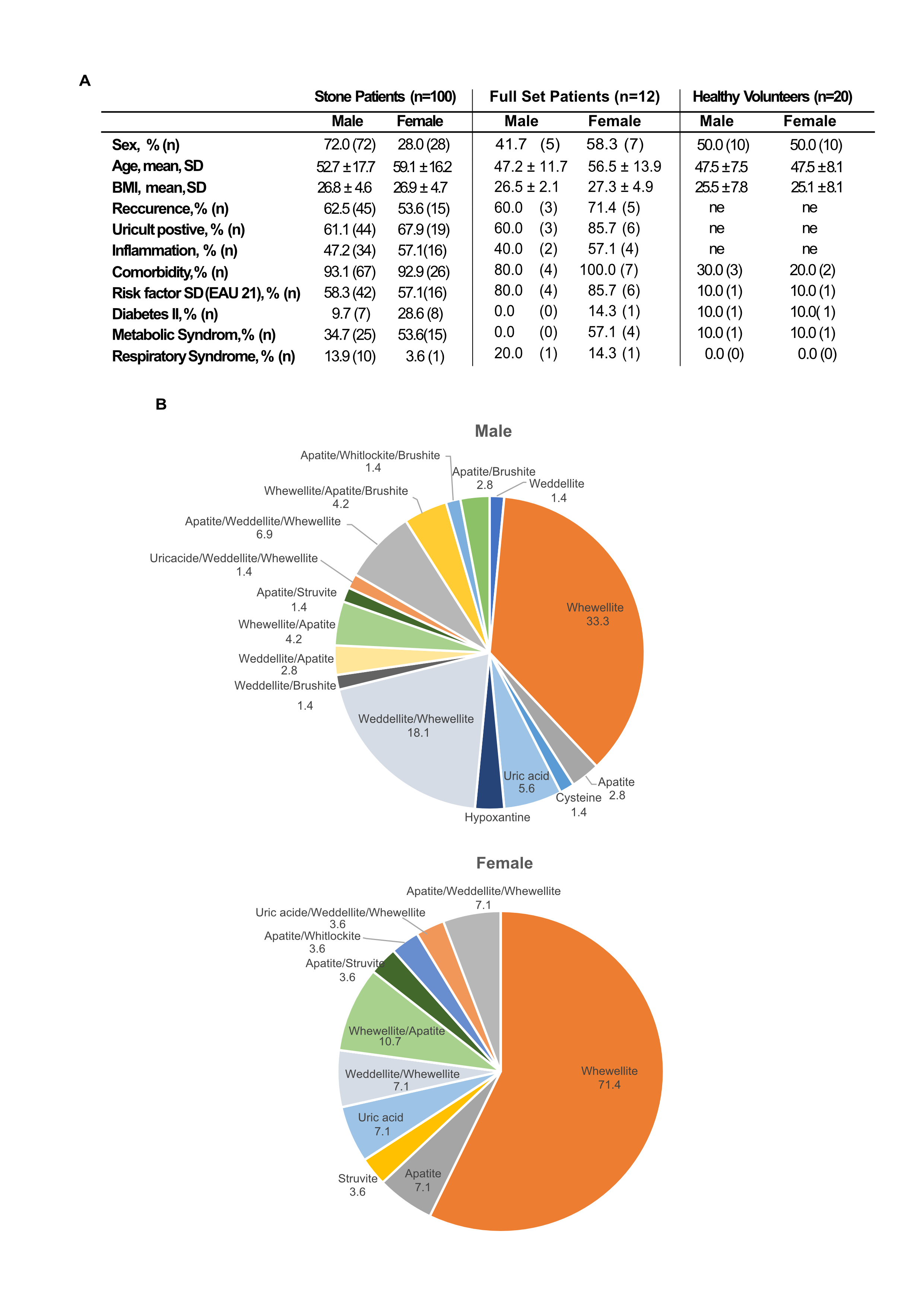

Supplement: Supplementary file 1 — Supplementary file1 Supplementary Fig. 1 Table with clinical data of all collected patients with nephrolithiasis and all patients with stone and urine sample >500 reads (A). Distribution of all collected stone types per sex in % (B) (TIFF 1100 KB) [file 240_2022_1403_MOESM1_ESM.tiff]

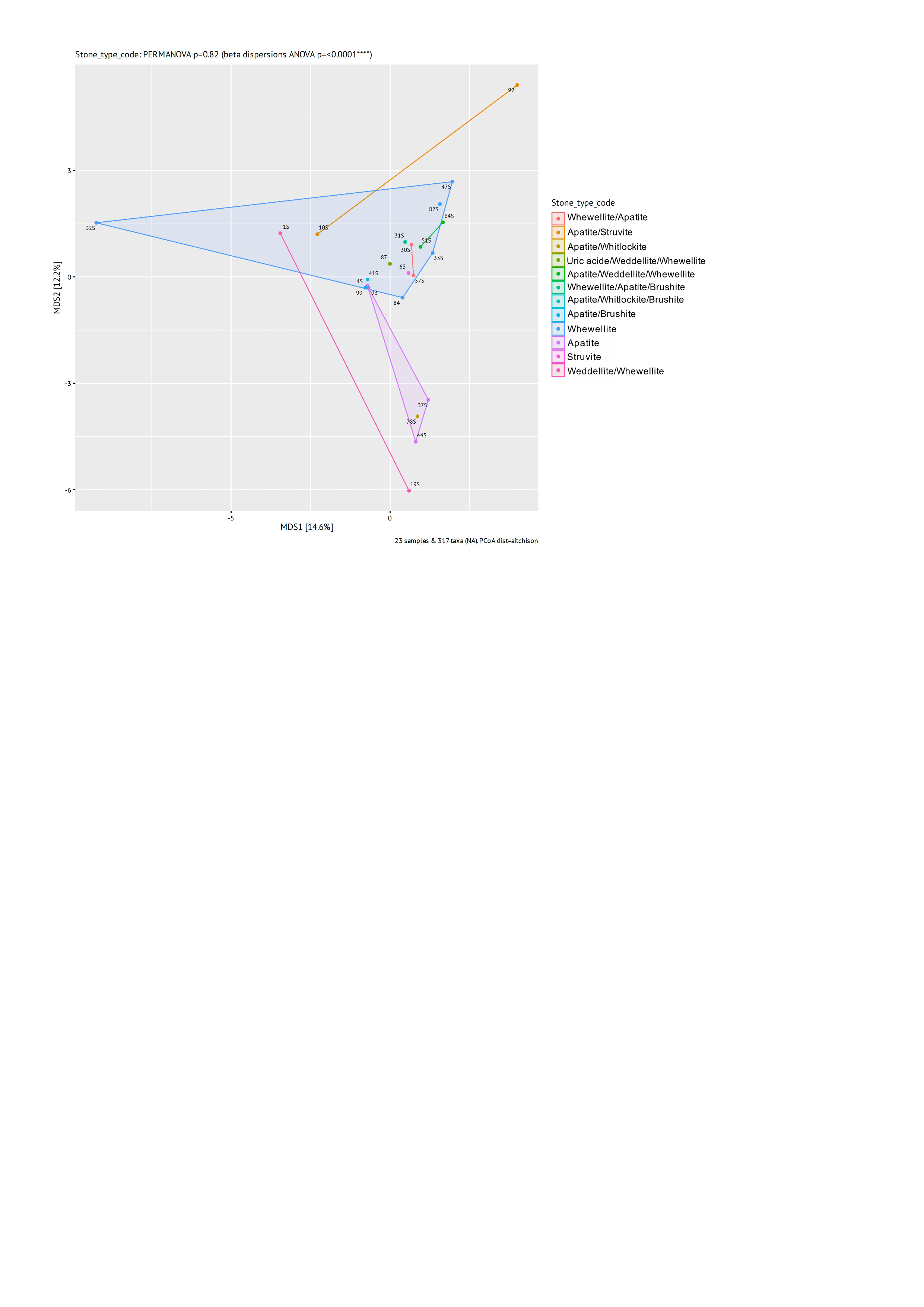

Supplement: Supplementary file 2 — Supplementary file2 Supplementary Fig. 2 PCoA of Stone type and associated microbiome. Kidney stones with the same chemical composition display no significant similarities in their microbiome (TIFF 688 KB) [file 240_2022_1403_MOESM2_ESM.tiff]

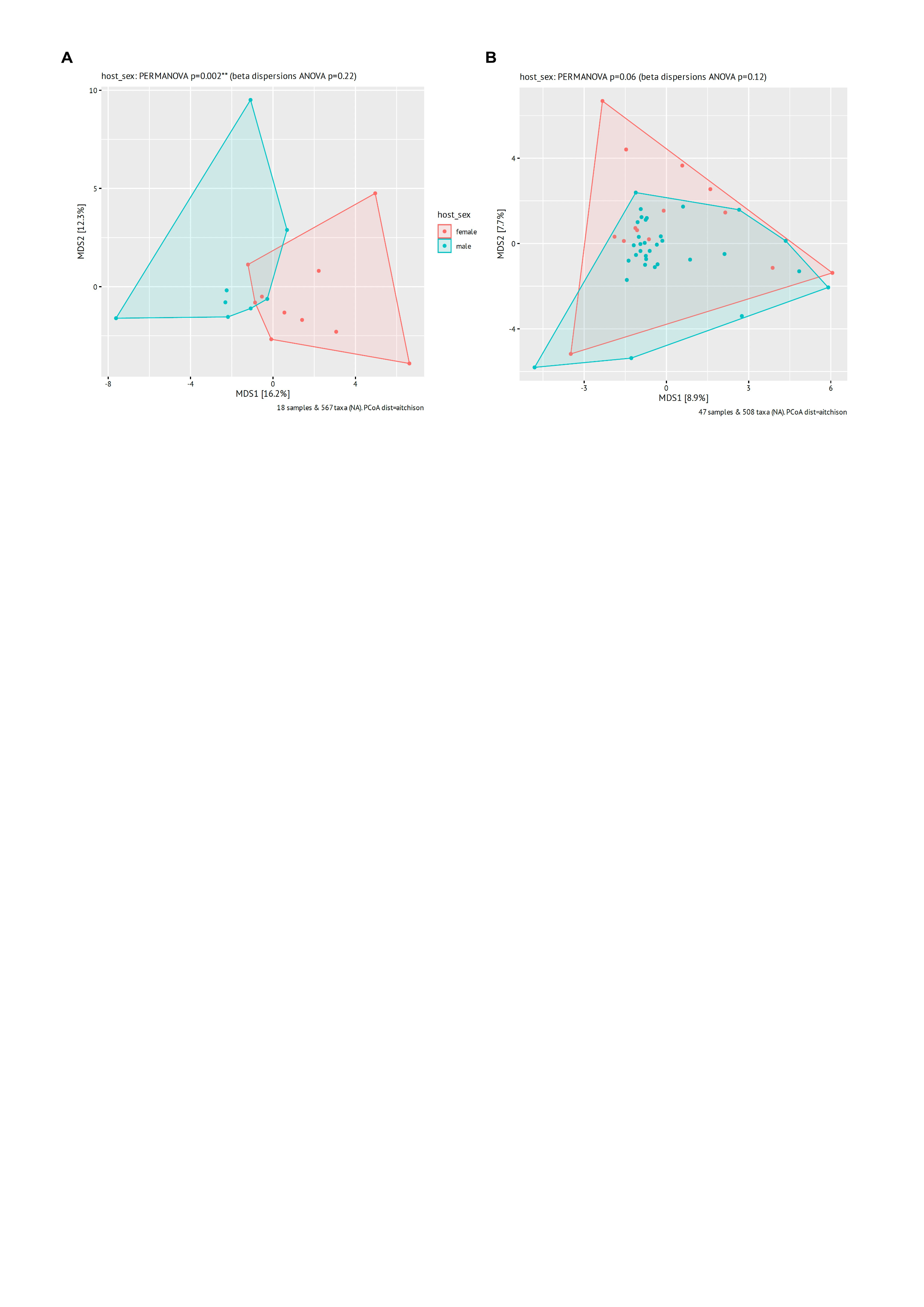

Supplement: Supplementary file 3 — Supplementary file3 Supplementary Fig. 3 Correlation of urine microbiome and sex. In men and women without stone disease urine samples displayed a significant difference in microbiome composition (A), while there is no difference between sexes in patients with nephrolithiasis (B) (TIFF 782 KB) [file 240_2022_1403_MOESM3_ESM.tiff]
